# Aqueous Fraction from *Hibiscus sabdariffa* Relaxes Mesenteric Arteries of Normotensive and Hypertensive Rats through Calcium Current Reduction and Possibly Potassium Channels Modulation

**DOI:** 10.3390/nu12061782

**Published:** 2020-06-15

**Authors:** Anas M.A. Alsayed, Bei Li Zhang, Pierre Bredeloux, Leslie Boudesocque-Delaye, Angèle Yu, Nicolas Peineau, Cécile Enguehard-Gueiffier, Elhadi M. Ahmed, Côme Pasqualin, Véronique Maupoil

**Affiliations:** 1 EA 7349, Laboratoire Signalisation et Transports Ioniques Membranaires; Groupe Physiologie des Cellules Cardiaques et Vasculaires, Faculté de Pharmacie, Université de Tours, 31 Avenue Monge, 37200 Tours, France; bei-li.zhang@univ-tours.fr (B.L.Z.); pierre.bredeloux@univ-tours.fr (P.B.); angele.yu@univ-tours.fr (A.Y.); nicolas.peineau@univ-tours.fr (N.P.); come.pasqualin@univ-tours.fr (C.P.); veronique.maupoil@univ-tours.fr (V.M.); 2 Department of Pharmacology, Faculty of Pharmacy, University of Gezira, Sudan. P.O. Box 20, 21111 Wad Medani, Sudan; yahadi54@yahoo.com; 3 EA 7502 SIMBA—Laboratoire Synthèse et Isolement de Molécules BioActives, Université de Tours, Faculté de Pharmacie, 31 Avenue Monge, 37200 Tours, France; leslie.boudesocque@univ-tours.fr (L.B.-D.); cecile.enguehard-gueiffier@univ-tours.fr (C.E.-G.)

**Keywords:** *Hibiscus sabdariffa*, hypertension, mesenteric artery, vasorelaxation, anthocyanins, Ca2+ current

## Abstract

Background/Objectives: *Hibiscus sabdariffa* L. (*H. sabdariffa* (HS)) extract has a vascular relaxant effect on isolated rat thoracic aorta, but data on small resistance arteries, which play an important role on the development of hypertension, are still missing. The purposes of this study were (1) to assess the effect on isolated mesenteric arteries (MA) from normotensive (Wistar and Wistar-Kyoto (WKY)) and spontaneous hypertensive rats (SHR); (2) to elucidate the mechanism(s) of action underling the relaxant effect in light of bioactive components. Methods: Vascular effects of HS aqueous fraction (AF) on isolated MA rings, as well as its mechanisms of action, were assessed using the contractility and intracellular microelectrode technique. The patch clamp technique was used to evaluate the effect of HS AF on the L-type calcium current. Extraction and enrichment of AF were carried out using liquid–liquid extraction, and the yield was analyzed using HPLC. Results: The HS AF induced a concentration-dependent relaxant effect on MA rings of SHR (EC_50_ = 0.83 ± 0.08 mg/mL), WKY (EC_50_ = 0.46 ± 0.04 mg/mL), and Wistar rats (EC_50_ = 0.44 ± 0.08 mg/mL) pre-contracted with phenylephrine (10 µM). In Wistar rats, the HS AF maximum relaxant effect was not modified after endothelium removal or when a guanylate cyclase inhibitor (ODQ, 10 µM) and a selective β2-adrenergic receptor antagonist (ICI-118551, 1 µM) were incubated with the preparation. Otherwise, it was reduced by 34.57 ± 10.66% when vascular rings were pre-contracted with an 80 mM [K+] solution (*p* < 0.001), which suggests an effect on ionic channels. HS AF 2 mg/mL significantly decreased the peak of the L-type calcium current observed in cardiac myocytes by 24.4%. Moreover, though the vasorelaxant effect of HS, AF was reduced by 27% when the nonselective potassium channels blocker (tetraethylammonium (TEA) 20 mM) was added to the bath (*p* < 0.01). The extract did not induce a membrane hyperpolarization of smooth muscle cells, which might suggest an absence of a direct effect on background potassium current. Conclusion: These results highlight that the antihypertensive effect of HS probably involves a vasorelaxant effect on small resistance arteries, which is endothelium independent. L-type calcium current reduction contributes to this effect. The results could also provide a link between the vasorelaxant effect and the bioactive compounds, especially anthocyanins.

## 1. Introduction

*Hibiscus sabdariffa* L. (*H. sabdariffa* L.) calyx has been used in many countries throughout the world as hot or cold beverages and for health purposes, especially for traditional treatment of high blood pressure [1,2,3].

Several clinical studies on *H. sabdariffa* have been carried out and have shown that the consumption of *H. sabdariffa* significantly lowers arterial blood pressure [4,5,6,7,8,9]. A number of in vivo pharmacological studies in experimental animals have also shown blood pressure lowering activity of *H. sabdariffa* [10,11,12]. Furthermore, *ex-vivo* pharmacological studies have documented that *H. sabdariffa* calyces extracts produce by vasorelaxation of the aorta and are either pre-contracted by noradrenaline, phenylephrine (PE), or depolarization with high potassium concentration [13,14,15,16]. Moreover, studies also showed that *H. sabdariffa* relaxes other smooth muscles, including the intestine, uterus, and bladder [17,18,19,20].

Several hypotheses have been suggested to explain the mechanism(s) involved in the reduction of arterial blood pressure, such as angiotensin-converting enzyme inhibition, the diuretic effect, or the direct vasorelaxant effect [21], but none have been clearly established. In some studies, it has been suggested that the relaxation is either endothelium dependent and/or endothelium independent due to inhibition of Ca^2+^ influx or activation of K^+^ channels [14,16,22], but no electrophysiological study has been performed to bring direct evidence.

Moreover, most of the vasorelaxant effect studies have been performed on a model of rat aorta, which is a capacitance artery poorly involved in the regulation of arterial blood pressure [23]. Therefore, experiments on resistance arteries that play an important role on the development of hypertension are still missing.

The active components that are responsible for the antihypertensive activity are not fully documented. However, anthocyanins are generally believed to be responsible for this action because they are found in high quantities in aqueous extracts [24,25,26].

Because of the folk medicine use of *H. sabdariffa* as a beverage, the main objectives of the study were (1) to investigate the effect of the aqueous fraction (AF) of *H. sabdariffa* on a model of resistance arteries (the mesenteric arteries from both hypertensive and normotensive rats). The spontaneously hypertensive rats (SHR) are commonly used as a model of human essential hypertension [27]. (2) to identify the mechanism(s) of action of *H. sabdariffa* AF in light of the phytochemical compound(s), especially anthocyanins.

## 2. Materials and Methods

### 2.1. Plant Materials

**Figure 1:**
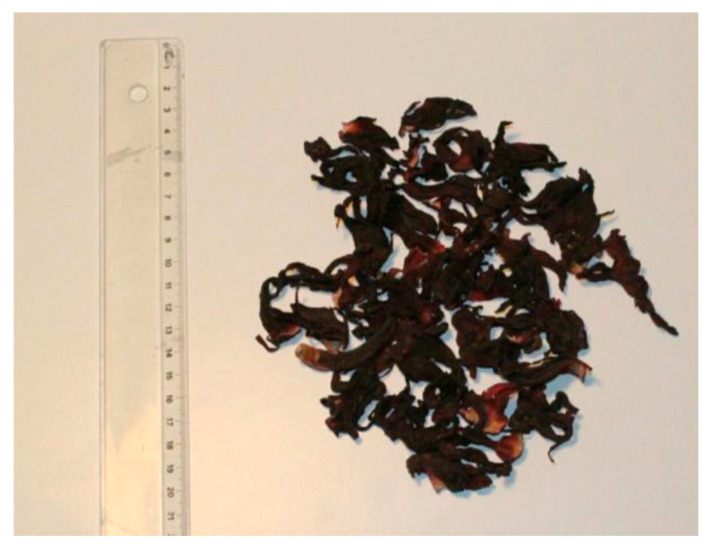
Herbarum photography of *Hibiscus sabdariffa*.

*Hibiscus sabdariffa* black-red dried calyces (*Malvaceae*), commonly known as Karkade, Roselle (Red Sorrel), or hibiscus, were procured from Gezira (Sudan) (Figure 1). The plant material was identified taxonomically by the Department of Pharmacognosy—University of Gezira number Hs-C/01. Botanical classification was checked with The Plant List (http://www.theplantlist.org) to get information on the folk medicinal use of the plant (accessed in July 2019).

### 2.2. H. Sabdariffa Extraction and Fractionation

For extraction, 500 g of coarse powdered crude dried calyces were macerated in 70% methanol in water (1:10, *w/v*) for 72 h at room temperature with occasional shaking and filtering. The filtrate was evaporated under vacuum to dryness (40 °C) and then freeze dried. For enrichment, 150 g of crude extract was dissolved in 20% ethanol in water (1:20 *w/v*), portioned with n-hexane (500 mL X 3) by liquid-liquid extraction using a separating funnel, followed successively and similarly by dichloromethane and ethyl acetate [16,25,28]. The remaining soluble fraction was used as an aqueous fraction, then, after filtration, the extract was concentrated under vacuum and freeze-dried. The AF was stored at −20 °C until further experiments.

### 2.3. HPLC Analysis

With the aim of confirming the chemical composition of the aqueous fraction, we proceeded to an HPLC analysis. The analysis was performed on a Dionex UHPLC U3000RS system equipped with a LPG-3400RS quaternary pump, a RSLC WPS-300T RS automated injector, a TCC-300SD column oven, and a UHPLC+ DAD-3000 diode array detector (ThermoFisher SA, Voisins le Bretonneux, France). The system was fitted with an Accucore aQ C18 (15 cm × 3 mm i.d., 2.6 µm particle size) column, itself protected by a Securiguard (Thermofisher). The mobile phases were solvent A 0.1% trifluoroacetic acid (TFA) in water and solvent B acetonitrile. The gradient was set as follows: The initial acetonitrile content was 0%, it was raised to 10% in 5.24 min, 15% in 0.32 min, 20% in 0.31 min, and 100% in 0.32 min and was maintained for 4 min. Flow rate was set at 0.8 mL/min, column temperature was set at 40 °C, and injection volume was 8 μL. A HPLC chromatogram from *Hibiscus* sabdariffa AF was recorded at 280 and 520 nm [29].

### 2.4. Animals

Experiments were performed on male Wistar, Wistar Kyoto (WKY), and spontaneously hypertensive rats (SHR) of about 16 weeks old, weighing between 390 to 515 g (CER Janvier, Le Genest, St Isle, France). All experiments involving animals were approved by the institutional ethical committee (Comité d’éthique en expérimentation animal Val de Loire, Tours, France, Permit number: 2016090711251954) and carried out in accordance with European guidelines on animal experimentation and the French “Ministère de l’Enseignement supérieur, de la Recherche et de l’Innovation”. All applicable international and/or institutional guidelines for the care and use of animals were followed. The blood pressure (BP) and heart rate (HR) of WKY rats and SHR were measured by use of the tail-cuff method [30].

### 2.5. Contractility Experiments on an Isolated Mesenteric Artery

All rats were anesthetized by intraperitoneal injection of pentobarbital (60 mg/kg). After intravenous injection of heparin (500 IU/kg), the mesenteric arterial bed was rapidly removed and placed into a dissecting dish that contained Krebs–Henseleit solution composed of (in mM) 119 NaCl, 25 NaHCO_3_, 4.7 KCl, 1.18 KH_2_PO_4_, 1.17 MgSO_4_, 1.36 CaCl_2_, and 5.5 glucose. All visible connective tissues were cleaned, and the second-order mesenteric arteries were cut into 1.5–2 mm ring segments and individually mounted in a Mulvany–Halpern myograph (Multi Myograph system 610 M, JP Trading, Aarhus, Denmark) for measurement of isometric tension. Vessels were perfused with a Krebs–Henseleit solution at a flow rate of 1 mL/min, maintained at 37 °C, and gassed with 5% CO_2_/95% O_2_. Data (mN/mm) were recorded with a PowerLab interface and Chart software (ADInstruments, Castle Hill, NSW, Australia). After a 30 min equilibration period in the Krebs–Henseleit solution, vessels were stretched (up to 3.5 mN) in a stepwise manner [31]. The rings were pre-contracted either with PE (10 µM) or high K^+^ solution (in mM, 39 NaCl, 25 NaHCO_3_, 80 KCl, 1.18 KH_2_PO_4_, 1.17 MgSO_4_, 1.36 CaCl_2_, and 5.5 glucose). Once the contractions reached the steady state, *H. sabdariffa* AF was perfused and the effect was recorded. In cumulative concentration-response curves, the vasoconstrictor responses were expressed as absolute contractions in mN/mm relative to basal tone. Relaxations were expressed as the percentage of the initial pre-contraction. Figures and curves, including EC_50_, were obtained from individual concentration-response curves. Confirmation of the presence of endothelium was demonstrated by a >30% relaxation in response to ACh (10 μM), when the mesenteric artery was pre-contracted with PE (10 μM). All the experiments were performed in the presence of endothelium, unless indicated. Otherwise, the endothelium was denuded by gently rubbing the luminal surface of the mesenteric artery rings.

In a separate set of experiments, the mechanism(s) of the vasorelaxant effect of *H. sabdariffa* AF were studied on mesenteric arteries isolated from Wistar rats using pre-incubation (20 min) and/or the presence of either a soluble guanylyl cyclase inhibitor (1*H*-[1,2,4] oxadiazolo [4,3,-*a*]quinoxalin-1-one (ODQ) 10 µM), selective β_2_-adrenergic receptor (adrenoreceptor) antagonist, (ICI-118551 (1 µM)), stable thromboxane A2 receptor agonist (U46619 (3 µM)), or a non-selective K^+^ channel blocker (tetraethylammonium chloride (TEA; 20 mM)). The rings were pre-contracted either with PE (10 µM) or U46619 (3 µM).

The controls were carried out for each experiment, either in the presence or absence of the agonist and/or the antagonist. At least three rats were used for each group of experiments. For all of these experiments, the pH of the physiological solution containing the extract was adjusted with NaOH up to 7.4.

### 2.6. Ventricular Cardiomyocytes Isolation

Cardiomyocytes isolation was proceeded, as mentioned previously [32]. Briefly, the heart and lungs were removed as one block from the anesthetized and heparinized rats and dissected in a cold (4 °C) cardioplegic solution bath (in mM: 110 NaCl, 16 KCl, 1.20 CaCl_2_, 16 MgCl_2_, 10 NaHCO_3_, and 10 glucose). Isolation was performed using a Langendorff system with 0.17 U/mL liberase™ in KRB (in mM: 35 NaCl, 4.75 KCl, 1.19 KH_2_PO_4_, 16 Na_2_HPO_4_, 25 NaHCO_3_, 134 sucrose, 10 HEPES, and 10 glucose, pH adjusted to 7.4) under 95% O_2_/5% CO_2_ until the heart changed in shape and color (white areas appeared). After enzymatic digestion, the heart was recovered in a KB solution (in mM: 50 glutamic acid, 30 KCl, 30 KH_2_PO_4_, MgSO_4_, 7H_2_O, 20 taurine, 0.5 EGTA, 10 HEPES, 10 Glucose, 10 KOH, pH = 7.4). The ventricles were then separated and cut up into fine pieces for mechanical digestion, then the KB solution was replaced by Tyrode’s solution (in mM: 140 NaCl, 5.37 KCl, 1.36 CaCl_2_, 1 MgCl_2_, 0.33 NaH_2_PO_4_, 10 HEPES, and 11 glucose; pH adjusted to 7.4) and stored at room temperature until use.

### 2.7. Whole Cell Patch-Clamp Experiments

The L-type calcium current (I_CaL_) was recorded from ventricular cardiomyocytes (VCMs) plated onto glass coverslips and maintained at room temperature (22–25 °C). During the patch clamp experiments, the cells were locally superfused by gravity (small capillaries positioned within 30 μm of the cell and allowing fast solution changes) with a solution containing (in mM): 144 TEACl, 2 CaCl_2_, 1 MgCl_2_, 5 4-aminopyridine, 10 HEPES buffer, and 11 glucose (pH 7.4 with TEAOH). The recording pipettes were pulled from borosilicate glass capillaries (Clark Electromedical Instruments) to a tip resistance of 2–4 MΩ. The pipette solution contained (in mM): 10 TEA-Cl, 2 MgCl_2_, 115 DL-aspartic acid, 5 di-*tris* phosphocreatine, 10 HEPES buffer, 10 EGTA, and 5 Mg-ATP (pH 7.25 with CsOH). These solutions were used to block ionic currents other than I_CaL_: K^+^ currents (TEA-Cl, CsCl, 4-aminopyridine, Na^+^ currents (Na^+^-free solutions), and Ca^2+^-activated currents (EGTA). An Axopatch 200 A amplifier connected to a PC computer, running Clampex (pClamp 9 software, Axon Instruments) through a Digidata 1200 A interface, was used to control voltage and record currents from a holding potential (HP) of −70 mV. Data were acquired at a sampling frequency of 10 kHz and low-pass filtered with an 8-pole lowpass Bessel filter at 2 kHz. Membrane capacitance (Cm) was measured by integration of the capacitive currents in response to a series of 10 hyperpolarizing pulses applied from the HP (amplitude and duration: 10 mV, 10 ms) and then averaged. The pipette and cell capacitances were compensated by 80% [32]. Maximum Ca^2+^ conductance (*g max*) and apparent reversal potential (*V rev*) were obtained from current-voltage curve (IV curve)fitting with the dedicated function:

(1) I=(V−V rev)×g max1+eV−V halfdV

where *V* half corresponds to the voltage at 50% of *g max*.

### 2.8. Microelectrode Experiments for the Measurement of Membrane Potential

The experiments were carried out as mentioned previously, with some modifications [33]. Mesenteric artery ring segments were taken from normotensive Wistar rats and individually mounted in a Mulvany–Halpern myograph. The myograph chamber was perfused using the Krebs–Henseleit solution at a flow rate of 5.4 mL/min, gassed with 95% O_2_/5% CO_2_ and maintained at 34 ± 0.1 °C. Resting membrane potentials was recorded with glass capillary microelectrodes, filled with 3 M KCl (30–50 MΩ), connected to a VF 180 microelectrode amplifier (Biologic). Voltage was recorded through a PowerLab interface and Chart software (ADInstruments, Castle Hill, NSW, Australia). Responses to *H. sabdariffa* AF were determined using a single dose (2 mg/mL), perfused for 20 min.

### 2.9. Data and Statistical Analysis

Analysis and presentation of data were performed by using Clampfit 10 and Origin 7 (Microcal Software, Northampton, MA, USA). Data were expressed as mean ± standard error of the mean (SEM) of results obtained from n different preparations. Statistical evaluation was performed with a Student’s *t*-test or a Mann–Whitney test. Differences were considered significant when *p*-values were less than or equal to 0.05.

### 2.10. Chemicals

Chemicals were obtained from either Merck KG (Darmstadt, Germany) or Sigma–Aldrich (Saint Quentin Fallavier, France), unless otherwise indicated. Solvents of extraction were from Carlo Erba (Val de Reuil, France). U46619 was from Cayman Chemical (Bertin Pharma, Montigny Le Bretonneux, France).

## 3. Results

### 3.1. Preparation of H. Sabdariffa AF and Characterization

**Figure 2:**
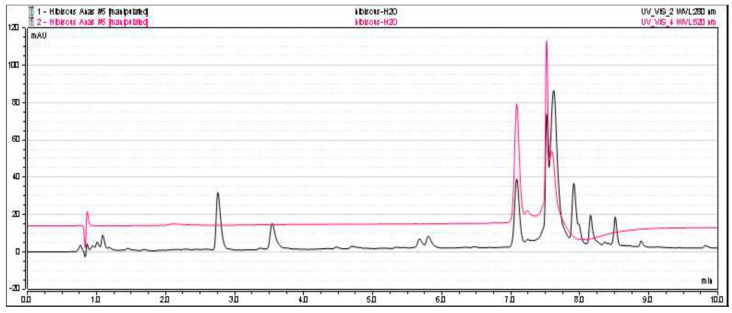
HPLC chromatogram from *H. sabdariffa* (HS) aqueous fraction (AF), recorded at 280 nm (black) and 520 nm (red).

The AF of *H. sabdariffa* (HS) was obtained using the classical extraction protocol [16,25,28]. The extraction yield of crude extract was 44.7%, and the resulting aqueous fraction represented 82% yield of the methanolic crude extract. HPLC analysis of *H. sabdariffa* AF showed the presence of two major peaks of anthocyanins at 520 nm, which is the commonly used wavelength for anthocyanin analysis [34]. At 280 nm HS, AF showed small peaks that represent the presence of a small amount of other phenolic/polyphenolic compounds. Therefore, aqueous fraction showed selectivity towards anthocyanins (Figure 2).

### 3.2. Effects of H. sabdariffa AF on Rat Mesenteric Artery Ring

**Figure 3:**
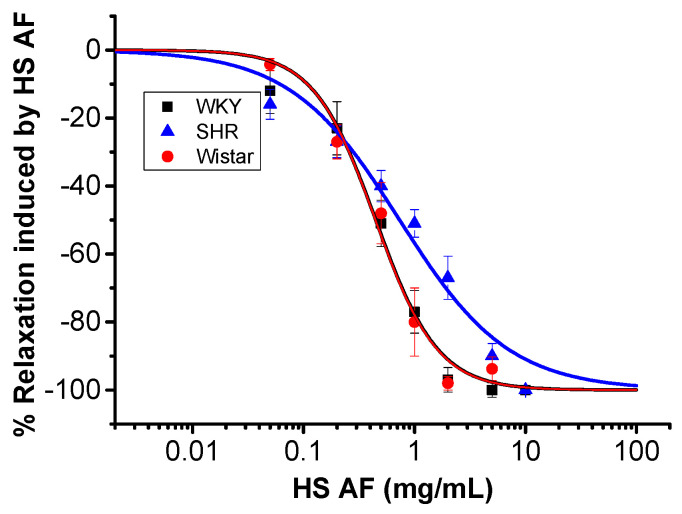
Concentration effect curve of HS AF on isolated mesenteric arteries (MA) rings of Wistar rat MA and WKY and SHR pre-contracted by 10 µM PE. Values are expressed as mean ± SEM (n = 9/5 for WKY, n = 8/4 for SHR, and n = 9/5 for Wistar). Relaxation was calculated as a percentage of the initial contraction.

In order to investigate the vasorelaxant activity of *H. sabdariffa* AF, mesenteric artery rings were isolated from male normotensive WKY and spontaneous hypertensive rats (SHR). The records of systolic BP, diastolic BP, and HR were significantly different between these two types of rats, and they were 195 ± 5.4, 151 ± 4.9 mmHg, and 347 ± 10.8 beats/min in SHR (n = 7), while they were 150 ± 4, 105 ± 7.4 mmHg, and 283 ± 4.5 beats/min in WKY rats (n = 6), respectively (*p* ˂ 0.05). Cumulative perfusion of different concentrations of *H. sabdariffa* AF (0.05–10 mg/mL) produced concentration-dependent relaxation of mesenteric artery rings isolated from both WKY rats (Emax = 98 ± 2% at HS AF 5 mg/mL) and SHR (Emax = 90 ± 3.7% at HS AF 10 mg/mL), pre-contracted by PE (10 µM). The effect of *H. sabdariffa* was not significantly different between SHR (EC_50_ = 0.83 ± 0.08 mg/mL, n = 8/4) and WKY rats (EC_50_ = 0.46 ± 0.04 mg/mL, n = 9/5) (*p* = 0.63) (Figure 3). In normotensive Wistar rat isolated mesenteric artery rings, *H. sabdariffa* AF (0.05–5 mg/mL) caused a concentration-dependent relaxation (Emax = 98 ± 2%, EC_50_ = 0.44 ± 0.08 mg/mL, n = 9/5, *p* = 0.09) that was not significantly different from that observed in WKY rats (Figure 3). Thus, these findings confirmed the vasorelaxant effect on mesenteric artery rings isolated from both normotensive and hypertensive rats.

### 3.3. Role of Endothelium and nitric oxide (NO) Pathway

**Figure 4:**
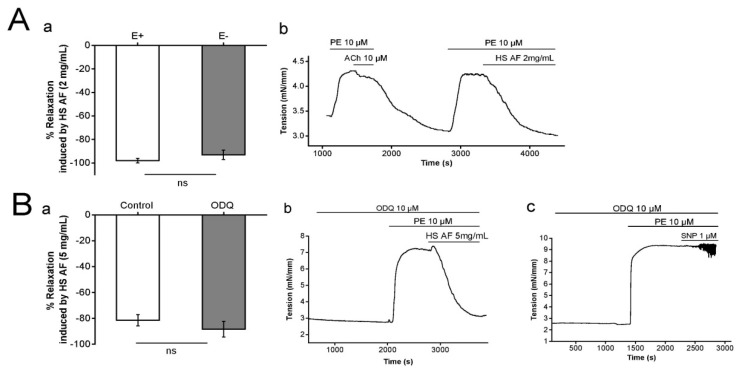
(**Aa**) Relaxant effect of 2 mg/mL HS AF on isolated Wistar rat MA ring pre-contracted by 10 µM PE in endothelium-intact (E+) and endothelium denuded (E−) rings. (**Ab**) Representative experimental trace of HS AF effect compared to 10 µM ACh-induced relaxation. Values are expressed as mean ± SEM (n = 8/4 for intact rings and n = 9/5 for denuded rings). (**Ba**) Effect of 5 mg/mL HS AF on isolated Wistar rat MA rings pre-contracted by 10 µM phenylephrine (PE) in presence and absence of 10 µM ODQ. (**Bb**) Representative experimental trace. MA rings were perfused with ODQ for 20 min before pre-contraction. (**Bc**) Control experiment by use of sodium nitroprusside (SNP) 1 µM. Values are expressed as mean ± SEM (n = 13/7 for control (PE + HS AF), n = 8/4 for 10 µM ODQ + (PE + HS AF)). Relaxation was calculated as a percentage of the initial contraction. Not statistically significant (ns).

The influence of the endothelium on the relaxant response of *H. sabdariffa* AF (2 mg/mL) was examined on Wistar rat isolated mesenteric artery rings pre-contracted by PE (10 µM). The endothelium intact rings showed vasorelaxation of 98 ± 2% (n = 8/4) and it was 93 ± 2% (n = 9/5) in endothelium denuded preparations. The maximum relaxant effect was not significantly decreased after endothelium removal of endothelium (*p* = 0.28) (Figure 4A). It suggests that endothelium is not involved in the vasorelaxant effect of AF on mesenteric artery rings.

Similarly, the possibility of nitric oxide (NO) donation by *H. sabdariffa* AF was tested by perfusion of ODQ, which is a selective, irreversible inhibitor of soluble guanylyl cyclase [35]. Our results showed that the vasorelaxant effect of *H. sabdariffa* AF was not affected by either the presence (88.4 ± 6.1%, n = 8/4) or absence (81.4 ± 4.4%, n = 13/7) of ODQ 10 µM (*p* = 0.38) (Figure 4Ba,b). In the control experiment, ODQ successfully blocked the effect of the NO donor sodium nitroprusside (SNP) 1 µM (Figure 4Bc), as expected. Hence, these results suggest that the vasorelaxation induced by *H. sabdariffa* AF was not due to NO donation. Therefore, the endothelium-independent effect of *H. sabdariffa* AF led us to study the probability of adrenergic pathway involvement.

### 3.4. Involvement of Adrenergic Pathway

**Figure 5:**
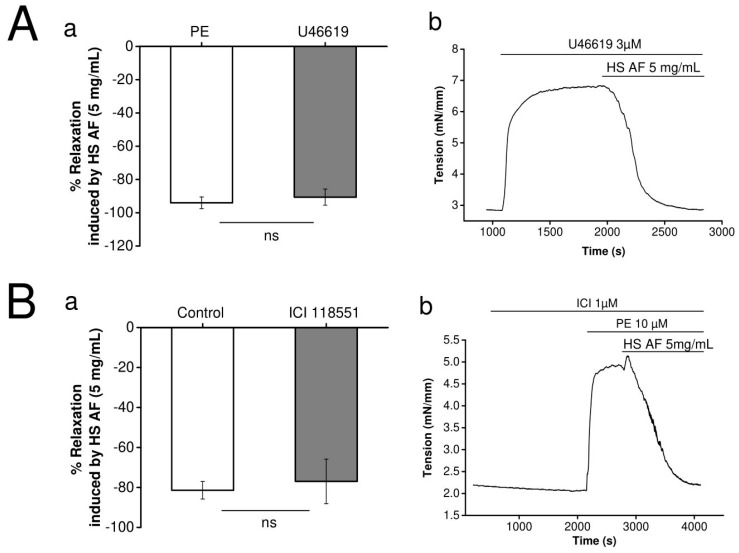
(**Aa**) Effect of 5 mg/mL HS AF on isolated Wistar rat MA rings in response to pre-contraction by 3 µM U46619 compared to the control. (**Ab**) Representative experiment trace. Values are expressed as mean ± SEM (n = 7/4 for control (PE + HS AF) and n = 8/4 for 3 µM U46619 + HS AF+). (**Ba**) Effect of 5 mg/mL HS AF with and without administration of 1 µM ICI 118,551 on isolated Wistar rat MA rings pre-contracted by 10 µM PE. MA rings were perfused using ICI 118551 for 20 min before pre-contraction. (**Bb**) Representative experimental trace. Values are expressed as mean ± SEM (n = 13/7 for control (PE + HS AF), n = 8/4 for 1µM ICI 118551 + (PE + HS AF)). Relaxation was calculated as a percentage of the initial contraction. Not statistically significant (ns).

In order to investigate the hypothesis that the extract has an α_1_-adrenergic receptor antagonist effect, *H. sabdariffa* AF (5 mg/mL) was tested on mesenteric artery rings pre-contracted by U46619 (3 µM), an agonist of the thromboxane A2 receptor, which is an another Gq pathway coupled receptor. As shown in Figure 5A, the vasorelaxant effect of *H. sabdariffa* AF was not significantly different when the artery was pre-contracted by 3 µM U46619 (90.6 ± 4.8%, n = 8/4) or by 10 µM PE (94 ± 3.5%, n = 7/4), *p* = 0.3 vs. PE pre-contraction.

Furthermore, we tested the hypothesis of the β_2_-adrenergic receptor agonism of *H. sabdariffa* AF on mesenteric artery rings pre-contracted by PE (10 µM). As presented in Figure 5B, perfusion of a selective β_2_-adrenergic receptor antagonist, ICI-118551 (1 µM), did not significantly (*p* = 0.4) attenuate the vasorelaxant response. It was 77.0 ± 11.1% (n = 8/4) and 81.4 ± 4.4% (n = 13/7) in presence and absence of ICI-118551, respectively.

These results indicate that neither α_1-_ nor β_2_-adrenergic receptors were involved in the vasorelaxant effect of *H. sabdariffa* AF. Therefore, we decided to investigate its direct effect on ionic currents.

### 3.5. Effect of H. sabdariffa AF on Ionic Currents

**Figure 6:**
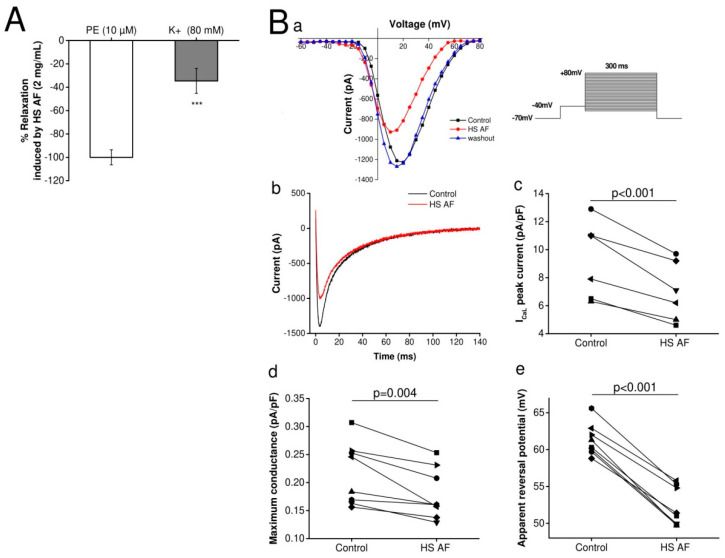
(**A**) Relaxant effect of 2 mg/mL HS AF on isolated Wistar rat MA rings pre-contracted by 10 µM PE or high K^+^ (80 mM) solution. Values are expressed as mean ± SEM (n = 9/5 for PE and n = 12/6 for high K^+^). Relaxation was calculated as a percentage of the initial contraction. *** *p* < 0.001 vs. 10 µM PE. (**B**) Effect of 5 mg/mL HS AF on the calcium current on isolated ventricular cardiomyocytes by use of the whole-cell voltage-clamp technique (n = 7/3). (**Ba**) I_CaL_ current-voltage curve in absence (black), in the presence of HS AF (red) and after washout (blue) (**Bb**)Representative traces of the calcium current recorded at +15 mV membrane voltage. . (**Bc**) Individual values of the maximum current recorded in the presence of HS AF and compared with control conditions. (**Bd**) I_CaL_ maximum conductance in the absence and presence of HS AF. (**Be**) I_CaL_ apparent reversal potential in the absence and presence of HS AF.

Wistar rat isolated mesenteric artery rings were pre-contracted with either PE (10µM) or high K^+^ solution, and the relaxant effects of *H. sabdariffa* AF at a concentration of 2 mg/mL were recorded for both experiments. The contraction induced by high K^+^ concentration is mainly due to an entry of calcium from extracellular space through the L-type calcium channels, whereas contraction induced by PE is mostly caused by the release of calcium from intracellular stores [36]. The results showed that *H. sabdariffa* AF relaxed mesenteric artery rings by 34.6 ± 10.7% (n = 12/6) when vascular rings were pre-contracted with high K^+^ solution, whereas the relaxation was 100 ± 6.42% (n = 9/5) when pre-contracted with PE (*p* < 0.001) (Figure 6A). The significant lower relaxant effect with high K^+^ pre-contraction suggests that *H. sabdariffa* AF might induce a partial effect on calcium entry from extracellular space. Therefore, further experiments on the calcium current were carried out using an electrophysiological technique.

According to the vasorelaxant effect of *H. sabdariffa* AF on mesenteric artery rings and the hypothesis of some authors, interestingly, we tested the effect of *H. sabdariffa* AF on the L-type calcium current using the whole-cell patch clamp technique on Wistar rat VCMs. The calcium current was activated by depolarizing voltage steps from −60 to +80 mV, with a prepulse at −40 mV (200 ms) to inactivate sodium current. The corresponding current voltage was obtained in a control condition, in the presence of *H. sabdariffa* AF, and after washout of *H. sabdariffa* AF to control the possible run-down of the calcium current [37]. *H. sabdariffa* AF induced a significant reduction of the calcium current (Figure 6Ba,b) of 24.4% (n = 7/3, *p* < 0.001) at the current peak (Figure 6Bc), and it was not due to I_CaL_ run-down, since the current fully recovered after washout of HS AF. The maximum conductance (*g max*) was reduced by 16.52 ± 0.03% (*p* = 0.004) (Figure 6Bd). These indicate that *H. sabdariffa* AF was able to decrease the L-type calcium current. Additionally, the apparent reversal potential (V rev) was shifted toward a lower voltage by 9.1 ± 0.6 mV (*p* < 0.001) (Figure 6Be), which suggests the increase of intracellular calcium concentration.

**Figure 7:**
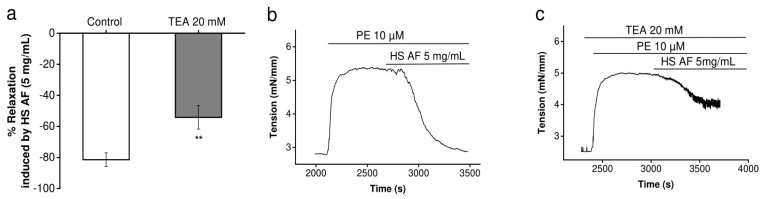
The effect of HS AF on potassium channels: (**a**) Effect of 5 mg/mL HS AF on isolated Wistar rat MA rings pre-contracted by 10 µM PE in the presence of 20 mM tetraethylammonium (TEA). MA rings were perfused using TEA for 20 min before pre-contraction. (**b**) and (**c**) Representative experimental traces in the presence and absence of TEA. Relaxation was calculated as a percentage of the initial contraction. Values are expressed as mean ± SEM (n = 13/7 for control (PE + HS AF) and n = 8/4 for TEA + (PE + HS AF). ** *p* < 0.01 vs. control (PE + HS AF).

Moreover, the significant lower relaxant effect of HS when vessels were pre-contracted with high K^+^ might also be explained by an effect on potassium channels. To establish whether the vasorelaxant effect of *H. sabdariffa* AF involves the activation of K^+^ channels, its effect was examined in the presence of the non-selective K^+^ channel blocker, tetraethylammonium chloride (TEA, 20 mM) on mesenteric arteries. using a Mulvany–Halpern myograph. The addition of TEA to the Krebs–Henseleit solution had no basal effect, but significantly decreased (27 ± 00%, *p* < 0.01) the vasorelaxation induced by *H. sabdariffa* AF, which could indicate that *H. sabdariffa* AF partially modulates the potassium channel (Figure 7).

**Figure 8:**
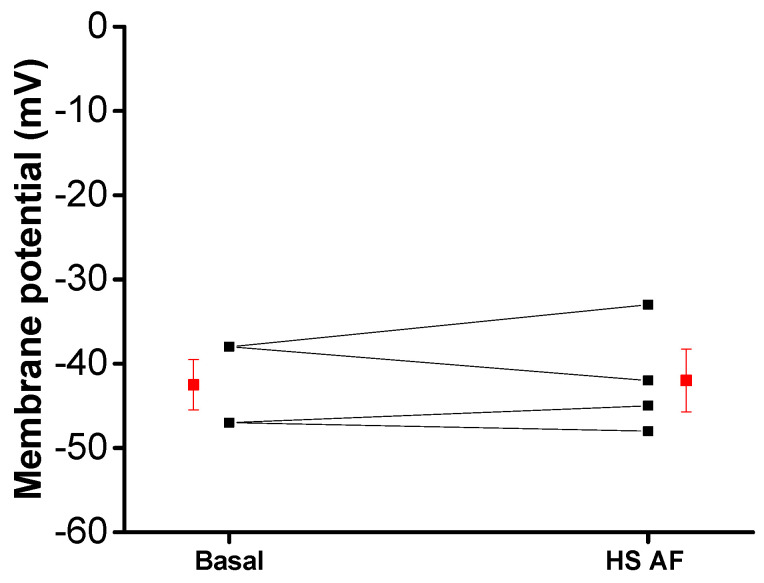
Effect of HS AF 2 mg/mL on the membrane potential of Wistar rat MA rings: individual (black) values and mean ± SEM (red) (n = 4/4).

Thereafter, in control conditions without PE, the membrane potential of smooth muscle cells in mesenteric artery rings was recorded using intracellular microelectrode before and after the addition of 2 mg/mL *H. sabdariffa* AF. The extract had no clear hyperpolarizing effect on the membrane potential of smooth muscle cells, which suggests that *H. sabdariffa* AF does not directly activate the potassium current (Figure 8).

## 4. Discussion

The objectives of this work were to demonstrate the relaxant effect of *H. sabdariffa* AF on the isolated mesenteric artery rings from normotensive and hypertensive rats, as well as to investigate the possible mechanism(s) of action in light of bioactive components that it contains.

Most of the vasorelaxation studies of *H. sabdariffa* were performed on a model of rat aorta, which is an elastic artery poorly involved in the regulation of arterial blood pressure compared to small resistance arteries [38,39]. In this regard, the results of the present study showed that the *H. sabdariffa* AF relaxed, concentration-dependently, the small mesenteric artery rings pre-contracted with PE (10 µM), whether they were isolated from normotensive or hypertensive rats. These findings are supported by the previous studies that demonstrated the action of *H. sabdariffa* extract [4,5,6,7,10,11,12,13,14,15,16,40,41].

In accordance with previous work that studied hibiscus acid, which is a water extract component of *H. sabdariffa*, on aorta [22], our results showed that there was no significant difference in relaxation achieved in either endothelium-intact or endothelium-denuded vessels. Therefore, the relaxation of mesenteric artery rings to *H. sabdariffa* AF seems to be endothelium-independent. This reinforces the hypothesis that a direct vasorelaxant effect is involved in the antihypertensive properties of *H. sabdariffa.* In contrast, previous studies using the crude hydro-alcoholic extract of *H. sabdariffa* found that the effect on aorta rings was reduced, but not lost, in the absence of endothelium [14,16], suggesting that there could be additional vasoactive components in the crude extract of *H. sabdariffa*.

Nevertheless, the present study suggests that the vasorelaxant effect of *H. sabdariffa* AF was neither due to an effect on the α_1_-adrenergic receptor, the β_2_-adrenergic receptor, nor activation of guanylyl cyclase. Therefore, the involvement of other mechanism(s) of action, particularly an action on ionic channels, was investigated.

**Figure 9:**
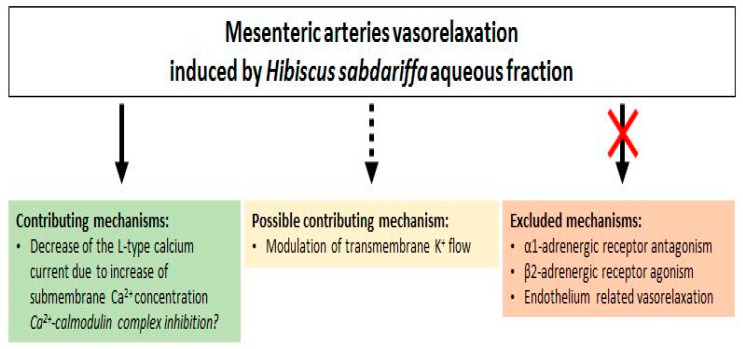
Diagrammatical representation of *H. sabdariffa* aqueous fraction mediated vasorelaxation in mesenteric arteries.

The pre-contraction with α_1_-adrenoceptor agonist (PE) and the depolarization with high K^+^ solution induces increases in smooth muscle tone by stimulating Ca^2+^ influx through voltage and receptor operated Ca^2+^ channels [36,42,43]. This might suggest that *H. sabdariffa* AF regulates these two Ca^2+^ entry pathways or, otherwise, its target is between Ca^2+^ and contractile proteins in the contraction signaling pathway. Previous studies suggested that the mechanism of action of *H. sabdariffa* might be associated with inhibition of Ca^2+^ influx through voltage-operated channels [14,15,40,44], but the direct effect of *H. sabdariffa* extract on the calcium current was not studied. For the first time, the whole-cell patch-clamp technique was used in this study to determine the effect of *H. sabdariffa* AF on the L-type calcium current. This experiment showed that *H. sabdariffa* AF partially decreases the L-type calcium current at a concentration, which induced the maximum vasorelaxant effect (Figure 9). However, this block is not great enough to explain the large vasorelaxant effect and probably contributes to only a part of this effect. Moreover, the reduction of the current is mainly due to the shift of the apparent reversal potential, rather than the decrease of the maximum conductance of the channels. This might be explained by an indirect effect of *H. sabdariffa* AF on this current, probably through the increase of sub-membrane calcium concentration. This observation suggests a possible inhibitory effect of the extract on the Ca^2+^-calmodulin complex [45].

Activation of potassium channels has also been proposed for the induction of vasorelaxation by the *H. sabdariffa* crude extract [16]. The preventing effect of TEA (non-selective K^+^ channels blocker) upon relaxation induced by our AF supports this hypothesis. Furthermore, the lower relaxant effect, when pre-contraction was induced by high concentration of external potassium compared to PE pre-contraction, could also support this mechanism; this is in agreement with that which was previously described in the aorta [14,40]. Indeed, the increase of extracellular potassium concentration reduces the driving force of K^+^ efflux and, therefore, decreases the possible hyperpolarization-induced vasorelaxant effect [42]. However, this mechanism could not be a direct opening of potassium channels, because microelectrode experiments have shown that *H. sabdariffa* AF did not induce a membrane hyperpolarization that would be expected by the increased outward potassium current in the basal condition. Hence, the mechanism might be an increase in a potassium current, activated after the pre-contraction (Figure 9).

The main bioactive constituents of *H. sabdariffa* relevant in the context of its pharmacological effects are polysaccharides, organic acids, and flavonoids, especially anthocyanins [46]. Generally, many flavonoids exhibit antihypertensive effects by targeting cardiovascular ionic channels, especially inhibition of calcium channels, activation of potassium channels, and/or Ca^2+^-calmodulin complex inhibition [47,48]. Thus, in order to highlight the link between this vasorelaxant effect and responsible phytochemical compounds, the aqueous fraction has been used in this study, which is the anthocyanins-rich fraction, besides some other phenolic/polyphenolic compounds, as shown in the phytochemical analysis. In this regard, a previous study documented that the maximum vasorelaxant effect on the aorta appeared with a *H. sabdariffa* fraction that contains mainly anthocyanins [16]. Furthermore, these findings are supported by previous in vivo studies and clinical trials, demonstrating the action of the aqueous extract of this plant [4,5,6,7,10,11,12]. It has been previously reported that *H. sabdariffa* contains various anthocyanins, mainly delphinidin-3-sambubioside (hibiscin) and cyanidin-3-sambubioside (gossypicyanin) [49,50]. It seems that the vasorelaxant activity would be due to the presence of these compounds, because they are detected in abundance in aqueous extracts of Hibiscus calyces [25,26,51]. Hence, these bioactive components can be a base for further investigations.

## 5. Conclusions

To the best of our knowledge, this study is the first report to provide data about the effect of *H. sabdariffa* on preparations of small resistance arteries, and it is the first report of an electrophysiological study highlighting the effect of the extract on the calcium current. Furthermore, the results of this study provide support for the possible use of *H. sabdariffa* in traditional medicine for hypertension and participate in the understanding of the mechanistic basis of the plant. Nevertheless, the total mechanism(s) is/are still not fully understood; therefore, further studies are required.

## Abbreviations

High-performance liquid chromatography	HPLC
Maximum effect	Emax
Concentration required to relax the induced tone by 50%	EC_50_
number of preparations/number of rats	n
Krebs-Ringer Bicarbonate Buffer	KRB
Hydroxyethyl piperazineethanesulfonic acid	HEPES
kraftbrühe	KB
